# Bioproduction, characterization, and evaluation of the biological activities of prodigiosin from *Serratia marcescens* HMS

**DOI:** 10.1007/s11274-026-04936-8

**Published:** 2026-04-25

**Authors:** Hadeer O. Jaheen, Hoda H. Yusef, Soraya A. Sabry, Mona E. M. Mabrouk

**Affiliations:** 1 Botany and Microbiology Department, Faculty of Science, Alexandria University, Alexandria, Egypt; 2 Botany and Microbiology Department, Faculty of Science, Damanhour University, Damanhour, Egypt

**Keywords:** *Serratia marcescens*, Intracellular and extracellular prodigiosin, Bioprocess optimization, Sustainable production, Characterization, Bioactivity evaluation

## Abstract

Prodigiosin is a bioactive red pigment with antimicrobial and anticancer properties, offering a natural substitute for synthetic colorants. A pigment-producing strain, *Serratia marcescens* HMS, was isolated from air and identified via 16S rRNA sequencing (GenBank Accession No. PP456732.1; 100% similarity). Acetone yielded the highest pigment extraction efficiency (4.54 mg/L). Optimal production (6.98 mg/L) occurred in LB medium (pH 7) at 30 °C under shaking conditions (150 rpm). Sweet potato peels served as an effective low-cost and sustainable agricultural waste substrate for extracellular pigment production. The purified pigment was confirmed as prodigiosin using TLC, HPLC, FTIR, and GC–MS (C_20_H_25_N_3_O; m/z 323). The pigment displayed antimicrobial activity against *Bacillus subtilis* (inhibition zone: 12 mm), cytotoxicity against HepG2 and MCF-7 cells (IC_50_: 61.12 ± 2.64 and 87.86 ± 4.18 μg/mL, respectively), and α-glucosidase inhibitory activity (IC_50_: 380.27 ± 8.15 μg/mL). These results establish *S. marcescens* HMS as an efficient prodigiosin producer and highlight the pigment’s potential for pharmaceutical and industrial applications.

**Graphical abstract:**

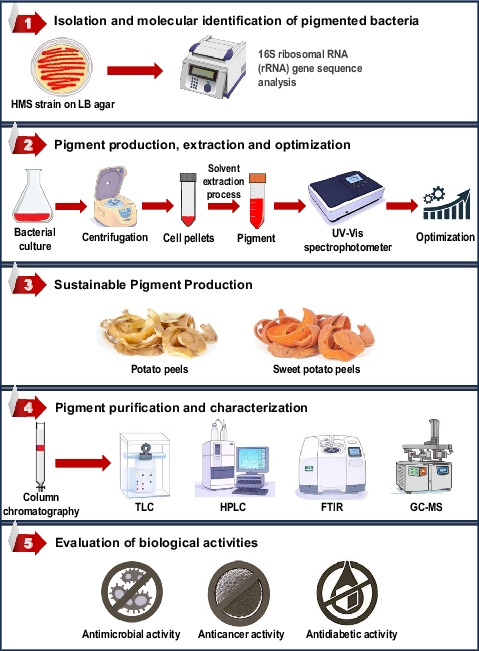

**Supplementary Information:**

The online version contains supplementary material available at 10.1007/s11274-026-04936-8.

## Introduction

Synthetic red dyes, including Allura Red AC (E129), Ponceau 4R (E124), and Erythrosine (E127), are widely used in food, cosmetics, pharmaceuticals, and textiles due to their color, stability, and low cost. However, growing evidence links these dyes to health risks such as hyperactivity, allergic reactions, DNA damage, and potential carcinogenicity. Notably, in 2025 the U.S. Food and Drug Administration revoked the authorization for FD&C Red No. 3 (erythrosine) in foods and ingested drugs due to cancer risks observed in animal studies, reflecting increasing regulatory scrutiny of synthetic dyes (Corradini 2019; Kim and Lee 2021; Mota et al. 2023; U.S. Food and Drug Administration. 2025). Beyond human health, synthetic dyes pose serious environmental challenges. Their non-biodegradable nature leads to contamination of waterways and soil, causing ecological disruption and long-term environmental hazards (Saini and Choudhary 2025).

These concerns highlight the urgent need for natural, safe, and sustainable alternatives, and microbial pigments have emerged as promising candidates. Microbial pigments are naturally produced by bacteria, fungi, and algae, and are generally biodegradable, non-toxic, and environmentally friendly. In addition to providing color, many microbial pigments possess bioactive properties such as antimicrobial, antioxidant, and anticancer activities (Rudrappa et al. 2023; Rather et al. 2025; Tang et al. 2025).

Prodigiosin is a red pigment primarily synthesized by *Serratia marcescens* and other microorganisms, exhibiting diverse bioactive properties. It functions as a potent anticancer agent against a variety of cancer cell lines, while showing minimal cytotoxicity toward normal cells. In addition to its anticancer activity, prodigiosin demonstrates antimicrobial, antioxidant, antiparasitic, algicidal, insecticidal, and immunomodulatory effects (Ehrenkaufer et al. 2020; Ma et al. 2025; Quintanilla-Villanueva et al. 2025; Rafiq et al. 2025; Zhang et al. 2025). The diverse bioactivities of prodigiosin make it a promising natural pigment with therapeutic potential, attracting extensive research attention in recent years.

Furthermore, microbial pigment production can be achieved using low-cost substrates, including agro-industrial waste, making the process environmentally friendly and economically feasible. This aligns with circular economy principles and offers potential for large-scale industrial application in pharmaceuticals, cosmetics, food coloring, and biomedical research (Deepika et al. 2025).

The present study focused on the production of prodigiosin, aiming to optimize both intracellular and extracellular yields while reducing overall production costs through the use of sustainable agro-waste substrates. While most previous studies have concentrated solely on intracellular prodigiosin. In addition, this research includes the purification, characterization, and evaluation of the pigment’s potential pharmaceutical applications.

## Materials and methods

### Isolation and molecular identification of the selected pigmented bacterial isolate

Airborne pigmented bacterial strains were isolated from a local radiology and imaging center and laboratories of Faculty of Science at Damanhour University. Nine LB agar plates were used for isolation: three placed at X-ray scanner, three at MRI device (magnetic resonance imaging), and three in a laboratory. The plates were exposed to air for 5 min, then immediately transported to Microbiology Laboratory. After incubating for 4 days at 30 °C, colored colonies were selected, purified by streaking, and checked for purity microscopically. Pure strains were maintained on LB slants at 4 °C. One intensely pigmented strain was selected for further study.

Genomic DNA was extracted from the selected bacterial isolate and used as a template for amplification of the 16S ribosomal RNA (rRNA) gene through polymerase chain reaction (PCR). Primers targeting a 1500 base pair region of the 16S rDNA were employed. The PCR amplification was carried out at Sigma Scientific Services Company, Cairo. Sequencing of the PCR amplicon was conducted by ACGT (DNA sequencing services company, Germany) using an ABI 3730xl DNA sequencer with universal primers 27F (5`-AGA GTT TGA TCC TGG CTC AG-3`) and 1492R (5`-GGT TAC CTT GTT ACG ACT T-3`). The obtained nucleotide sequence was subjected to similarity search using the Basic Local Alignment Search Tool (BLAST) available at the National Center for Biotechnology Information (NCBI; http://www.ncbi.nlm.nih.gov/BLAST/). Identification of the isolate was based on sequence homology with reference strains in the NCBI database. For phylogenetic analysis, a tree was generated using MEGA version 11 software to determine the evolutionary relationship with closely related taxa. The partial 16S rRNA gene sequence was submitted to NCBI GenBank, and an accession number was assigned (Poddar et al. 2021).

### Pigment production, extraction with different solvents and quantification

HMS strain was grown in LB broth for 7 days at 30 °C with shaking at 120 rpm. To determine the most effective solvent for extracting intracellular pigments, a variety of polar solvents [ethanol, methanol, acetone, ethyl acetate, dimethyl sulfoxide (DMSO), acetone: methanol (1:1 v/v), and acetone: ethanol (1:1 v/v)] as well as non-polar solvents (hexane and chloroform) were evaluated. Five milliliters of culture were centrifuged at 4000 rpm for 10 min to collect cell pellets, which were washed with distilled water and recentrifuged. Pellets were then resuspended in an equal volume of solvent, vortexed for 1 min, and centrifuged again at 4000 rpm for 10 min. This process was repeated until the pellets lost their color. The crude pigment was collected and stored at 4 °C for further use (Usman et al. 2018).

The absorbance spectrum of the extracted intracellular pigment was measured in the range of 200 to 800 nm by C-7200A UV–Vis Spectrophotometer. Intracellular pigment production was quantified as absorbance at 535 nm using UV–Vis spectrophotometer, prodigiosin concentration (Mahmoud et al. 2015) and pigmentation degree of cells (Mal et al. 2020).

The extracellular pigment was evaluated by measuring the absorbance of the culture supernatant at 535 nm using a UV–Vis spectrophotometer.

$$\text{Prodigiosin concentration}\left(\mathrm{mg}/\mathrm{L}\right)=\frac{{OD}_{535} \times MW}{{E}_{535}\times L}\times 1000\times DF$$

where:

OD_535_: Optical density at 535 nm
MW: Molecular weight of prodigiosin = 323.4 g/mol
E_535_: 7.07×10^4^ M^-1^ cm^-1^ (Molar extinction coefficient of prodigiosin at 535 nm)
L: Cuvette path length = 1 cm
DF: Dilution factor = $\frac{final\ volume}{Sample\ volume}$  

$$\text{Degree of cell pigmentation}=\frac{{OD}_{\lambda max}}{{OD}_{600}}$$

where:

OD_λmax_: Optical density at the maximum absorption wavelength
OD_600nm_: Optical density at 600 nm of the culture

### Growth and pigment production kinetics

Pigment production along with growth kinetics were determined by growing *S. marcescens* HMS in LB broth at 30 °C with shaking (120 rpm). Culture samples (5 mL) have been collected every 24 h, and the growth was evaluated by determine the optical density at 600 nm. The intracellular pigment had been extracted from the cell biomass after the culture samples were centrifuged. Pigment yield and growth were plotted against time.

### Optimization of pigment production

One-variable-at-a-time method (Hagaggi and Abdul-Raouf 2023) was used to determine the optimum parameters of pigment production by *S. marcescens* HMS. The effect of different parameters i.e., culture media (LB broth (Bertani 1951), nutrient broth (Chauhan and Jindal 2020), yeast malt medium (Otero et al. 2019), and synthetic medium (Shatila et al. 2013)), incubation temperature (25, 30 and 35 °C), pH of culture medium (3,4,5,6,7,8,9 and 10) and shaking rates (0, 120 and 150 rpm) affecting pigment production, was evaluated. The growth and the intracellular and extracellular pigment yields were estimated.

### Sustainable pigment production using agricultural wastes

Potato and sweet potato peels were used to evaluate their effect on intra- and extracellular pigment production and cell growth. The samples were obtained from local market, and the peels were rinsed with tap water before being dried for 24 h at 60 °C in a hot air oven. The peels were dried, grounded to powder, and then stored in sterile containers. One gram of each peel powder was placed in each 100 mL Erlenmeyer flask containing 20 mL of distilled water. The mixture was allowed to soak for 5 min, followed by shaking and filtration (Adomi and Oyubu 2023). Following sterilization, 1 mL of seed culture was introduced into each flask and incubated at 30 °C with rotary shaking at 120 rpm for 7 days.

### Pigment purification and characterization

The pigment was produced at optimized conditions and extracted as previously mentioned. All pigmented supernatant was collected and concentrated using rotary evaporator at 40 °C to dryness, and the crude pigment was obtained and stored at 4 °C. Crude extracts were subjected to fractionation via silica gel column chromatography. A glass column (1.5 cm in diameter and 50 cm in length) was packed with 20 gm of silica gel (G100; particle size 63–200 μm), following the insertion of a stopper at the lower tapered end. The silica gel was pre-activated by heating at 60 °C for 3 h. The extract was separated using open-column chromatography, employing a stepwise elution protocol (10-mL volume each) beginning with five column volumes of petroleum ether, followed by a mixture of petroleum ether and ethyl acetate (1:1 v/v), continued until the red-colored fraction was eluted, as described by Bhagwat and Padalia (2020). Fractions were collected individually and stored at 4 °C. The red eluate, containing purified prodigiosin, was concentrated using rotary evaporator at 40 °C.

**Thin Layer Chromatography (TLC):** Each active fraction was subjected to TLC to assess purity. Selected fractions exhibiting similar TLC profiles were combined accordingly. TLC analysis was conducted using aluminum-backed plates precoated with silica gel G-60 (GF_254_, 0.2 mm thickness; Merck, Darmstadt, Germany). Sample application was performed automatically with the CAMAG® LINOMAT 5 sample applicator. Chromatographic separation was carried out using a solvent system of acetone: hexane (7:3 v/v) ratio (Bhagwat and Padalia 2020). Following development, TLC plates were air-dried at room temperature, and purity analysis was conducted using the CAMAG® TLC Scanner. Vitamin A served as the reference standard for compound comparison. Retention factor (Rf) values were calculated using the subsequent equation (Gupta et al. 2019) and were compared with previously reported values of other literatures.

$$\text{Retention factor}\left(\mathrm{Rf}\right)=\frac{Distance\ travelled\ by\ the\ compound}{Distance\ travelled\ by\ the\ solvent}$$

**High Performance Liquid Chromatography (HPLC):** HPLC analysis was conducted using an Agilent 1220 Infinity LC system equipped with a ZORBAX SB-C18 column (4.6 × 250 mm, 5 μm; Agilent Technologies). Both the purified pigment extract and the standard prodigiosin (Cat#529685, CALBIOCHEM, Germany) were prepared in methanol at a concentration of 1 mg/mL. An aliquot (3 μL) of each sample was injected for analysis. Chromatographic separation was achieved using a mobile phase consisting of methanol: water: acetonitrile in a ratio of 73:20:7, at a constant flow rate of 0.8 mL/min. The column temperature was maintained at 30 °C, and detection was performed at a wavelength of 535 nm using a UV detector (Song et al. 2006; Tran et al. 2021).

**Fourier Transform Infrared Spectroscopy (FTIR):** FTIR spectra of the dried and purified red pigment was performed using a Nicolet 5700 FTIR spectrometer. The sample was prepared by thoroughly grinding the dried pigment with potassium bromide (KBr) to obtain a fine powder, which was then compressed into pellets using a KBr press (ATLAS T25, ATS). Spectral analysis was conducted at room temperature over the wavenumber range of 400 to 4000 cm⁻^1^ (Paul et al. 2024).

**Gas Chromatography and Mass Spectrometry (GC–MS):** Five µL of the pure pigment were injected into the GCMS system after it had been dissolved in 1 mL of methanol (Bhagwat and Padalia 2020). Chromatographic analysis was performed using a Trace GC1310-ISQ Mass Spectrometer (Thermo Scientific, Austin, TX, USA) equipped with a TG-5MS capillary column (30 m × 0.25 mm, 0.25 µm film thickness). The column oven temperature was initially set at 35 °C and increased at a rate of 3 °C per minute up to 200 °C, where it was held for 3 min. This was followed by a further increase to 280 °C at the same rate, with a final hold time of 10 min. The temperatures of the MS transfer line and injector were kept at 260 °C and 250 °C, respectively. Helium served as the carrier gas at a constant flow rate of 1 mL/min. Following a 3-min solvent delay, automated injection of 5 µL of the diluted sample was carried out using the AS1300 Autosampler in split mode. Mass spectra were acquired in full scan mode with electron ionization (EI) at 70 eV, covering a mass-to-charge (m/z) range of 40–1000. The ion source temperature was maintained at 200 °C.

### Evaluation of biological activity of prodigiosin

#### Antimicrobial activity

Six bacterial and fungal strains obtained from the Regional Center for Mycology and Biotechnology, Al-Azhar University – Cairo, Egypt were used in the current study. The antibacterial and antifungal activities of the pigment were tested against several bacterial strains, including Gram-negative bacterial species (*Escherichia coli* ATTC 25922, *Proteus vulgaris* RCMB 004 (1) ATCC 13315), and Gram-positive bacterial species (*Staphylococcus aureus* ATTC 25923, *Bacillus subtilis* RCMB 015 (1) NRRL B-543), as well as against fungi including a mold (*Aspergillus fumigatus* RCMB 002008) and a yeast (*Candida albicans* RCMB 005003 (1) ATTC 10231). The tests were carried out using a modified well diffusion method (Abo-Ashour et al. 2018; Rasras et al. 2023). A volume of 100 μL of each test bacterium or fungus was cultured in 10 mL of fresh growth medium (Müeller-Hinton broth for bacteria and Sabouraud Dextrose broth for fungi) until reaching approximately 10⁸ CFU/mL for bacteria and 10^5^ CFU/mL for fungi. Subsequently, 100 μL of each microbial suspension was uniformly spread onto solid agar plates prepared with their respective media. Wells of 6 mm diameter were aseptically punched into the agar, and each was filled with 100 μL of the purified pigment solution (10 mg/mL). Plates were then incubated at 37 °C for 24–48 h for bacteria and yeast, and at 28 °C for 48 h for filamentous fungi. Following incubation, microbial growth was assessed by measuring the diameter of the inhibition zones in millimeters, which served as an indicator of antimicrobial activity. DMSO employed as the pigment solvent was used as a negative control and showed no inhibitory activity. Gentamycin (4 μg/mL) and ketoconazole (100 μg/mL) served as positive controls for bacteria and fungi, respectively. All tests were performed in triplicate.

#### Anticancer activity

**Cell lines:** Human breast adenocarcinoma (MCF-7) and human hepatocellular carcinoma (HepG2) cell lines were obtained from the American Type Culture Collection (ATCC, Rockville, MD, USA). The cells were maintained in RPMI-1640 medium supplemented with 10% inactivated fetal calf serum and 50 µg/mL gentamicin. The cultures were incubated at 37 °C in a humidified atmosphere containing 5% CO₂ and routinely subcultured twice to three times per week (Mosmann 1983; Abo-Ashour et al. 2019).

**Cytotoxicity evaluation using viability assay:** MCF-7 and HepG2 tumor cells were seeded at 5 × 10^4^ cells/well in Corning® 96-well tissue culture plates and incubated for 24 h. Tested pigment was added in triplicate wells across twelve concentrations. Six wells per plate were treated with 0.5% (v/v) DMSO as a negative control, and Cisplatin was included as a positive control. Cell viability was assessed using MTT assay following 24 h of exposure to the test compounds. Following incubation, the culture medium was replaced with 100 µL of phenol red–free RPMI-1640 medium, and 10 µL of 12 mM MTT solution (5 mg/mL in phosphate-buffered saline) was added to each well, including controls. Plates were incubated for 4 h at 37 °C in 5% CO₂. Subsequently, 85 µL of medium was removed and 50 µL of DMSO was added to solubilize the formazan crystals. Plates were mixed thoroughly and incubated for an additional 10 min at 37 °C. Absorbance was measured at 590 nm using a microplate reader (SunRise, TECAN, Inc., USA).

Cell viability was calculated as a percentage using the formula:

$$\%\mathrm{Viability}=\left({\mathrm{OD}}_{\mathrm{t}}/{\mathrm{OD}}_{\mathrm{c}}\right)\times 100$$

where OD_t_ is the mean optical density of treated wells, and OD_c_ represents the mean optical density of untreated control wells.

Survival curves were generated by plotting the percentage of viable cells against pigment concentration for each cell line. The 50% inhibitory concentration (IC_50_), indicating the concentration required to reduce cell viability by 50%, was calculated using nonlinear regression analysis with GraphPad Prism software (Mosmann 1983; Abo-Ashour et al. 2019).

#### Antidiabetic activity

The in-vitro antidiabetic potential of prodigiosin was assessed via α-glucosidase inhibition assay, employing BioVision’s α-Glucosidase inhibitor screening kit (K938-100). Prodigiosin was dissolved in DMSO and tested across twelve different concentrations, each assayed in triplicate wells. For each reaction, 10 µL of prodigiosin solution was combined with 10 µL of α-glucosidase enzyme solution diluted 1:20 in assay buffer according to the kit protocol, and 10 µL of the substrate 4-nitrophenyl-α-D-glucopyranoside (PNPG) in a 96-well microplate. The mixture was incubated at room temperature for 15–20 min. Positive control wells contained Acarbose, enzyme control (EC) wells contained assay buffer only, solvent control (SC) wells contained DMSO without test compound, and background control (BC) wells lacked enzyme. Enzymatic hydrolysis of PNPG by α-glucosidase yields p-nitrophenol, a chromogenic product detectable at 410 nm. The reaction was terminated by adding 50 µL of 0.2 M sodium carbonate. Absorbance at 410 nm was measured for both test and blank samples. ΔOD/Δt slopes were calculated and blank- and solvent-corrected by subtracting BC and SC values. A p-nitrophenol (PNP) standard curve was prepared to quantify enzymatic activity.

$$\begin{aligned}&\%\text{Relative inhibition was calculated as}:\%\text{ Inhibition}\\&=\frac{Slope \ of \ EC-Slope \ of \ Sample}{Slope \ of \ EC}\times 100\end{aligned}$$

Using GraphPad Prism software, the 50% inhibitory concentration (IC₅₀) of prodigiosin was calculated through graphic plots.

### Statistical analysis

All experimental data are presented as the mean ± standard deviation (SD) from three independent replicates (*n* = 3). Statistical analysis was conducted using IBM SPSS software (version 31). Differences among groups were evaluated by one-way analysis of variance (ANOVA), and *p*-values ≤ 0.05 were considered statistically significant.

## Results

### Isolation and molecular identification of pigmented bacteria

**Fig. 1:**
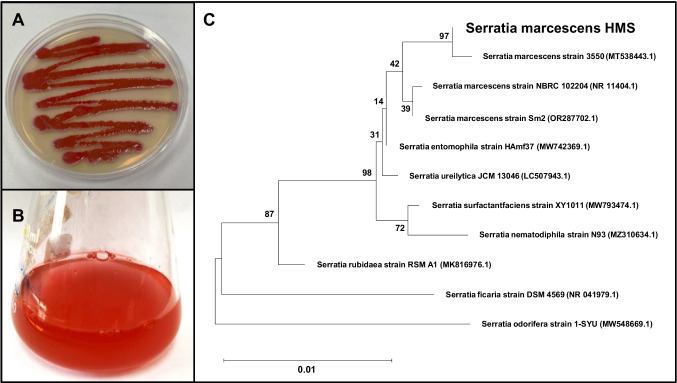
HMS strain grown on solid and liquid media (**a**, **b**). Phylogenetic tree demonstrating the relationship between *S. marcescens* HMS and related members of *Serratia* based on 16S rRNA sequence (**c**)

Eleven pigmented bacterial strains were isolated from the air using LB medium. Among these, four strains produced yellow pigments, six produced orange pigments, and one produced a red pigment. The red pigmented isolate was chosen for further study because of its intense pigmentation and was named HMS (Fig. 1a and b). The purified HMS bacterial isolate was preserved on LB slants and stored at 4 °C.

HMS was identified through 16S rRNA gene sequencing. The results revealed that HMS shared 99–100% similarity with several strains of *Serratia* spp. The isolate was confirmed to be *Serratia marcescens* and was designated as *Serratia marcescens* HMS (Fig. 1c). The 16S rRNA sequence has been submitted to GenBank with the accession number PP456732.1.

### Effect of different solvents on pigment extraction

**Fig. 2:**
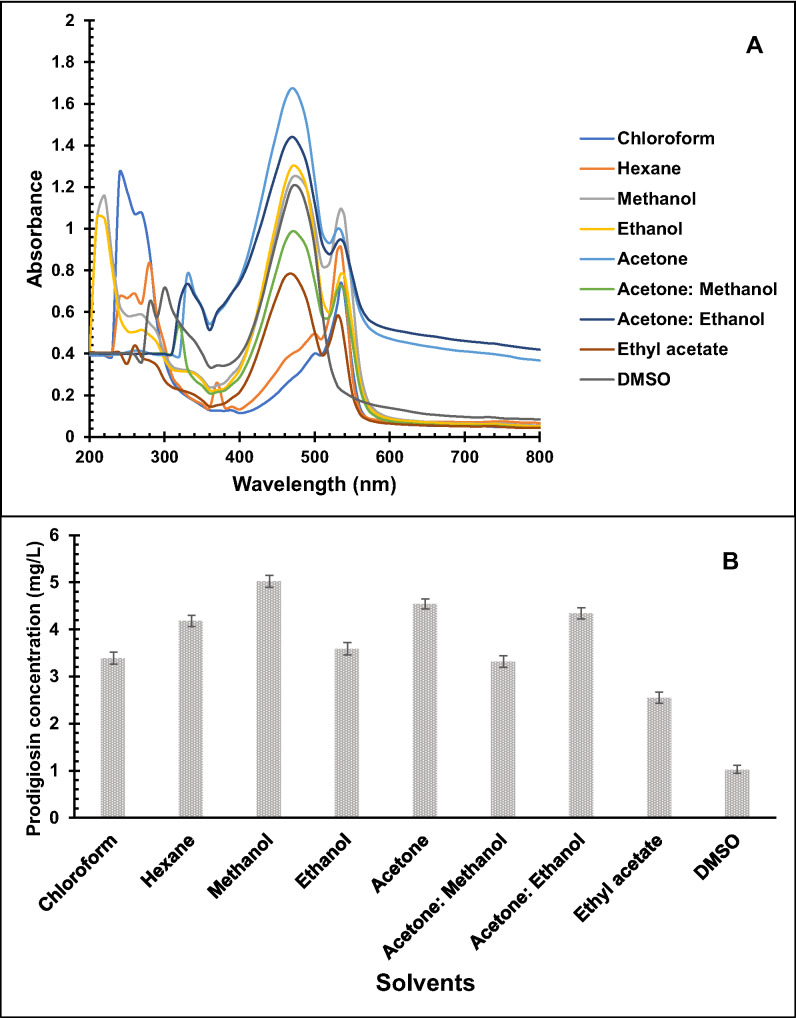
UV–Vis spectra of intracellular prodigiosin extracted from *S. marcescens* HMS using different solvents (**a**), Effect of different solvents on the concentration of intracellular prodigiosin extracted from *S. marcescens* HMS (**b**). Bars represent the mean prodigiosin concentration (± SD, *n* = 3) for each solvent. One-way ANOVA demonstrated a significant difference in prodigiosin yield among the solvents tested (*p* < 0.001)

The pigment extracted from *S. marcescens* HMS was analyzed using UV–Vis spectrophotometry across a range of 200–800 nm with various solvents to determine its λ_max_ (Fig. 2a). The spectrum revealed peak absorbance at 470 nm and 535 nm, similar to the UV–Vis spectrum of the prodigiosin pigment. The UV–Vis spectra of non-polar solvents like chloroform and hexane did not display the characteristic peak at 470 nm, while DMSO lacked the peak at 535 nm. The solvent extraction process identified methanol and acetone as the most effective solvents for extracting the intracellular pigment, with pigment yields differing significantly among solvents (*p* < 0.001). The prodigiosin pigment yield extracted using methanol was estimated at 5.02 mg/L, with absorbances of 1.245 and 1.097 at 470 nm and 535 nm, respectively. Extraction with acetone yielded 4.54 mg/L with absorbances of 1.675 and 0.992 at 470 nm and 535 nm, respectively (Fig. 2b). Therefore, acetone is a highly sensitive and effective solvent for pigment extraction, particularly due to its lower toxicity compared to other solvents. Additionally, its lower boiling point than methanol allows for faster processing times. As a result, acetone was determined to be the optimal choice for extracting prodigiosin pigment from *S. marcescens* HMS.

### Growth and pigment production kinetics

The kinetics of growth and pigment production for *S. marcescens* HMS, as shown in Fig. S1, indicated a relationship between pigment production and cell growth. Cell growth entered the stationary phase after 72 h of cultivation, while the highest prodigiosin concentration was reached after 96 h of incubation. Statistical analysis revealed that both growth and pigment production varied significantly over time (*p* < 0.001).

### Optimization of pigment production

**Fig. 3:**
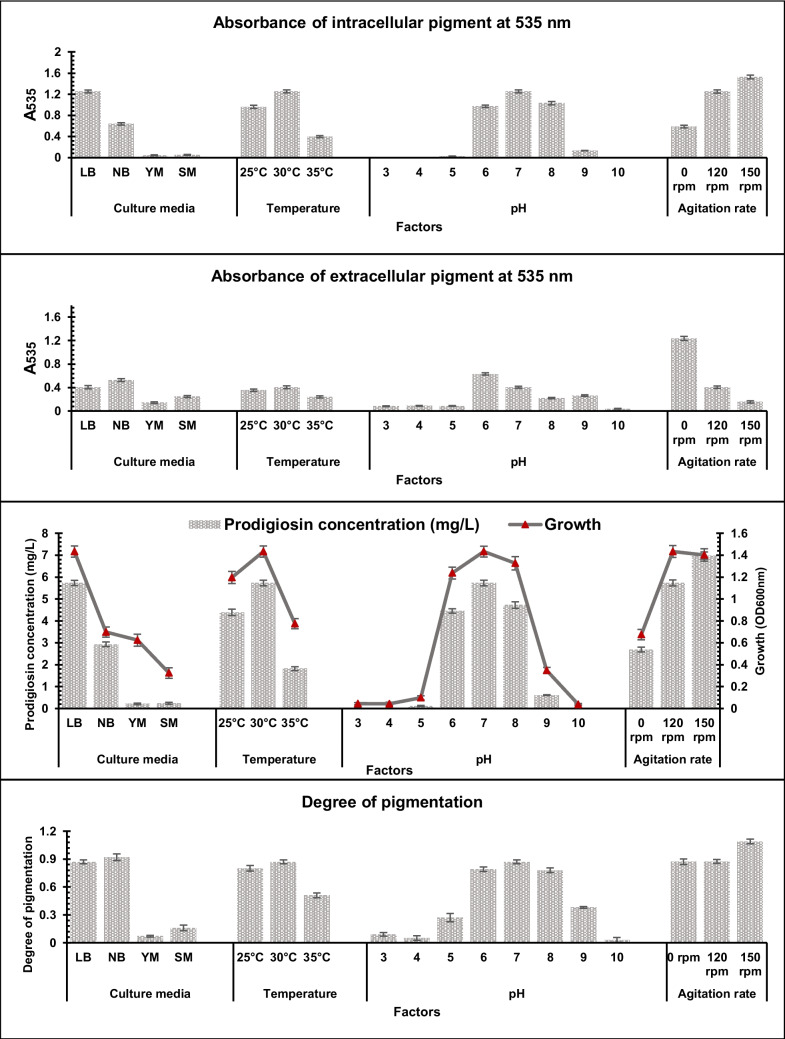
Optimization of pigment production by *S. marcescens* HMS. Bars represent the mean values (± SD, *n* = 3) for all tested parameters. Statistical significance was assessed for prodigiosin concentration using one-way ANOVA (*p* < 0.001)

Environmental and culture conditions have a direct impact on both the pigment biosynthesis and the bacterial growth (Rana et al. 2021). The effects of several conditions, including culture media, temperatures, pHs, and shaking rates, on *S. marcescens* HMS growth and pigment production were examined in the present study. The results showed that the highest pigment production occurred under the same conditions that promoted optimal growth (Fig. 3). The ideal conditions for both growth and pigment production were found to be the cultivation of *S. marcescens* HMS on LB broth (pH 7) in shaken condition (150 rpm) at 30 °C (Table S1).

Pigment concentration was significantly influenced by media composition, temperature, pH, and agitation rate (*p* < 0.001), indicating that all tested factors had a strong effect on pigment production.

**Fig. 4:**
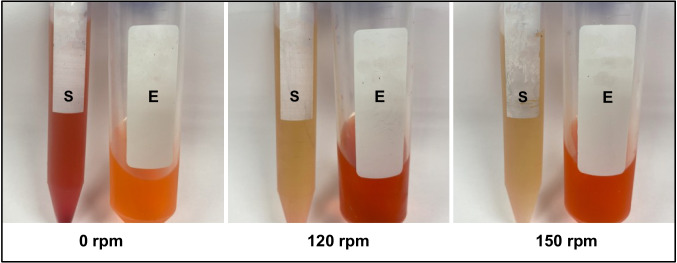
The effect of static and shaking conditions on prodigiosin production by *S. marcescens* HMS (E: extracted intracellular pigment, S: culture supernatant)

A comparison of the pigment production at static and different shaking conditions (Fig. 4) revealed that shaking increased intracellular pigment production. On the other hand, extracellular pigment increased at static condition. Additionally, cell growth was greatly enhanced in the shaking cultures, with biomass reaching nearly twice the level observed in static conditions.

### Sustainable pigment production using agricultural wastes

Peels of potato and sweet potato were used as substrate for the cultivation of *S. marcescens* HMS. The intracellular pigment showed no absorbance at 535 nm. On the other hand, the culture supernatant exhibited high absorbance value of 2.025 and 1.354 at 535 nm, indicating the extracellular production of prodigiosin using sweet potato peels and potato peels, respectively as a sole carbon and nitrogen sources (Fig. S2). For comparison, the highest extracellular pigment production in LB medium under static conditions showed an absorbance of 1.236 at 535 nm. Further analysis is required to optimize prodigiosin production by *S. marcescens* HMS using these substrates.

### Pigment purification and characterization

The crude pigment extract was purified according to the previously established protocol, and the resulting red fractions were subjected to a comprehensive set of characterization techniques.

Vitamin A, a compound with a pyrrole ring system analogous to prodigiosin and a comparable molecular weight, was employed as a reference standard. Fractions 5 and 6, along with their combination, were applied to a TLC plate, where the observed Rf values were 0.86, 0.87, and 0.86, respectively (Fig. S3). The extracted red pigment exhibited Rf values consistent with those of vitamin A (0.85). The Rf values were determined to be close to the range of 0.87–0.89, which aligns with the reported characteristics of prodigiosin.

The purified red pigment exhibited a retention time comparable to that of the standard prodigiosin, as shown in Fig. S4. HPLC analysis revealed that both the purified sample and the standard produced a single peak, with retention times of 5.06 min and 5.20 min, respectively, indicating similarity in their chromatographic profiles.

**Fig. 5:**
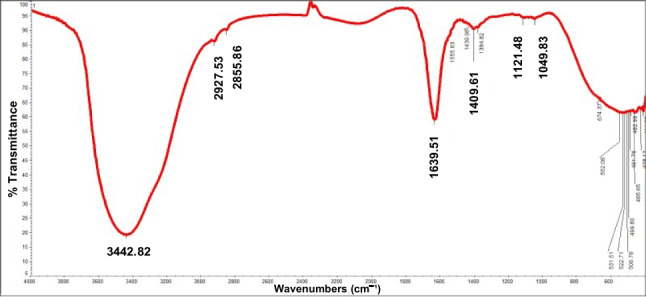
The FTIR spectrum of the purified red pigment

The FTIR spectrum of the purified red pigment (Fig. 5) reveals broad and strong absorption peak at 3442 cm^−1^ (O–H and N–H stretch), two weak absorption peaks at 2927 cm^−1^ and 2855 cm^−1^ (C-H and C = O stretches), and a strong absorption peak at 1639 cm^−1^ (C = N stretch) which indicates the presence of pyrrolenine. Furthermore, there are weak absorption peaks at 1409 cm^−1^ (C-H bend), 1121 cm^−1^ and 1049 cm^−1^ that show (C-N bend) and (C-O stretch) which indicates amines and carboxylic presence, respectively.

**Fig. 6:**
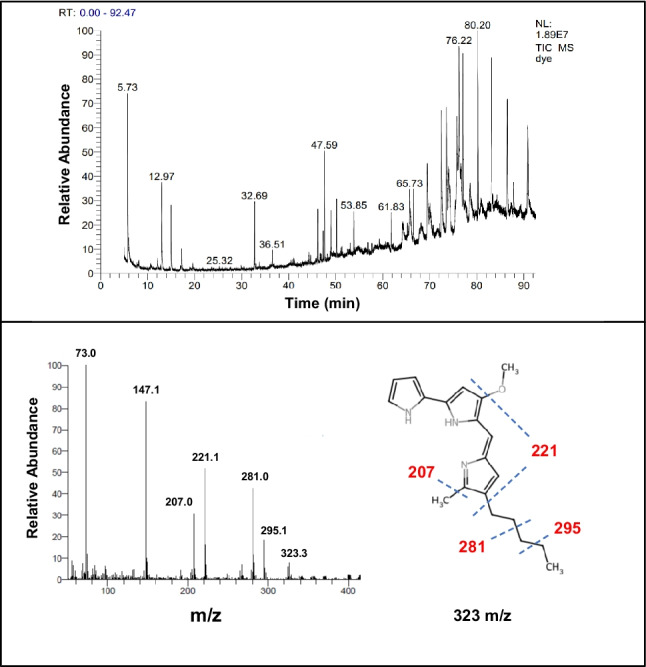
GC–MS analysis of the extracted pigment and its derivatives

GC–MS analysis of the purified red pigment revealed 36 distinct major peaks at different retention times (Fig. 6). The mass spectrum showed a molecular ion peak at 323 m/z, consistent with prodigiosin (C_20_H_25_N_3_O). Figure 6 illustrates the mass spectrum and chemical structure of prodigiosin, along with its characteristic fragment ions. The detailed GC–MS profile of purified prodigiosin, including retention times, relative peak areas, and major fragment ions, is presented in Table S2.

In summary, the TLC, HPLC, FTIR, and GC–MS results consistently confirmed that the purified red pigment corresponds to prodigiosin, as evidenced by comparable Rf values, similar retention times, characteristic functional groups, and a molecular ion peak at 323 m/z.

### Evaluation of biological activity of prodigiosin

#### Antimicrobial activity

**Fig. 7:**
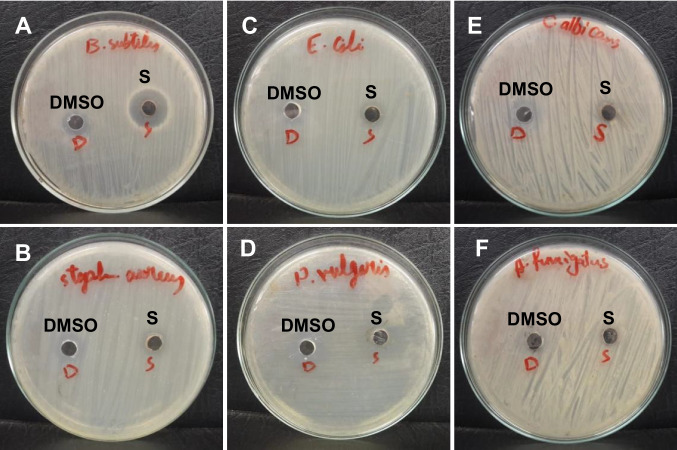
The antimicrobial activity of pure prodigiosin against *Bacillus subtilis* (**a**), *Staphylococcus aureus* (**b**), *Escherichia coli* (**c**), *Proteus vulgaris* (**d**), *Candida albicans* (**e**), and *Aspergillus fumigatus* (**f**), (DMSO: dimethylsulfoxide as a negative control, S: prodigiosin sample)

Purified prodigiosin pigment extracted from *S. marcescens* HMS demonstrated an inhibitory effect against the Gram-positive *Bacillus subtilis*, as shown in Fig. 7 and Table S3, with an inhibition zone diameter of 12 mm. However, no inhibitory activity was observed against the other tested microorganisms.

#### Anticancer activity

**Fig. 8:**
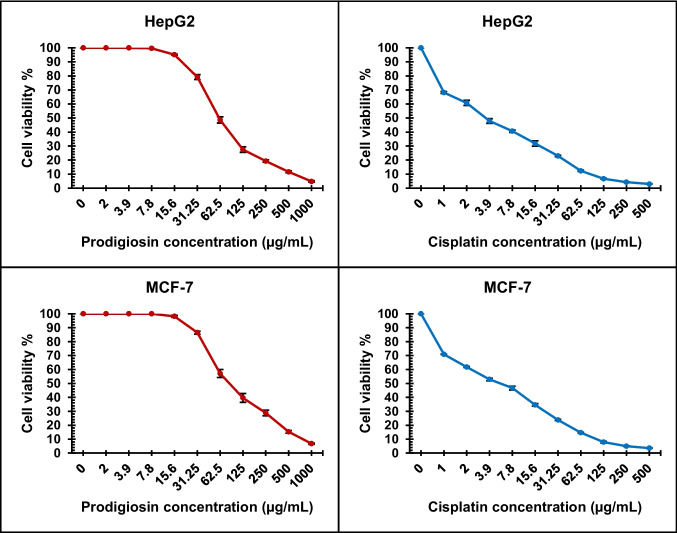
Inhibitory activity of prodigiosin pigment and cisplatin against viability of HepG2 and MCF-7 cells. Values are presented as mean ± SD (*n* = 3). One-way ANOVA showed significant differences in cell viability among treatments (*p* < 0.001)

The MTT assay was utilized to assess the cytotoxic activity of purified prodigiosin produced by *S. marcescens* HMS in vitro against different cancer cell lines. In the current study, DMSO and the chemotherapeutic agent cisplatin served as negative and positive controls, respectively. Chemosensitivity was evaluated by generating concentration response curves, as shown in Fig. 8. The results demonstrated that prodigiosin had significant inhibitory effects on the viability of HepG2 and MCF-7 cells (*p* < 0.001). The calculated inhibitory concentration of 50% (IC_50_) for HepG2 cells was 61.12 ± 2.64 µg/mL, while for MCF-7 cells, the IC_50_ was 87.86 ± 4.18 µg/mL. At a concentration of 1000 µg/mL, prodigiosin achieved maximum inhibition levels of 95% and 93% for HepG2 and MCF-7 cells, respectively. In contrast, cisplatin reached maximum inhibition levels of 97% and 96% for HepG2 and MCF-7 cells, respectively, at a lower concentration of 500 µg/mL.

#### Antidiabetic activity

**Fig. 9:**
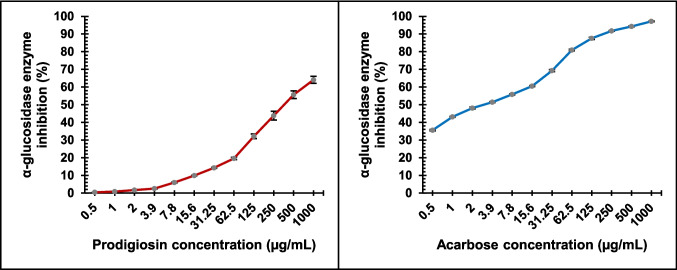
The α-glucosidase inhibitory activity of prodigiosin pigment and acarbose. Values are presented as mean ± SD (*n* = 3). One-way ANOVA showed significant differences in α-glucosidase inhibition among treatments (*p* < 0.001)

Prodigiosin showed a significant inhibitory effect on α-glucosidase (*P* < 0.001), with a maximum inhibition of 64% observed at a concentration of 1000 µg/mL. In contrast, acarbose achieved a higher maximum inhibition of 97% at the same concentration (Fig. 9). The IC_50_ values for prodigiosin and acarbose were calculated as 380.27 ± 8.15 µg/mL and 3.13 ± 0.19 µg/mL, respectively.

Collectively, purified prodigiosin exhibited antibacterial activity against *Bacillus subtilis*, significant cytotoxic effects against HepG2 and MCF-7 cell lines, and moderate α-glucosidase inhibitory activity, confirming its biologically active properties.

## Discussion

Biosafety assessments of prodigiosin are essential for evaluating its potential applications in pharmaceutical and biomedical fields. Prodigiosin extracted from *S. marcescens* ZPG19 demonstrated a beneficial effect on the intestines of Kunming mice, where administration was associated with improvements in intestinal microbiota composition and overall gut health. Importantly, no toxic effects were observed in the internal organs of treated mice, indicating an acceptable in vivo safety profile (Li et al. 2021). While these results are encouraging, it is important to emphasize that current evidence is largely limited to early-stage in vivo studies.

This study aimed to explore pigmented bacterial isolates from natural and radiation-exposed air environments to enhance the selection of pigment-producing strains (Khan et al. 2024). Among the isolates obtained, one strain exhibiting red pigment production was identified as *S. marcescens* HMS based on 16S rRNA gene sequence analysis.

The UV–Vis spectral analysis confirmed that the pigment extracted from *S. marcescens* HMS is prodigiosin, as evidenced by its characteristic absorption peaks at 470 nm and 535 nm. These results align with previous findings (Fu et al. 2019), further supporting the identification of the pigment as prodigiosin. Although methanol yielded a slightly higher pigment concentration, acetone demonstrated higher absorbance at 470 nm and offers several practical advantages. Its lower toxicity and lower boiling point compared to methanol allow for faster and safer extraction processes, making it more suitable for large-scale or sensitive applications (Lababpour and Lee 2006; Park et al. 2012). This is supported by multiple reports identifying methanol, ethanol, and acetone as the most effective solvents for prodigiosin extraction (Arivizhivendhan et al. 2015; Paul et al. 2024). Therefore, acetone was selected as the optimal solvent for further extraction of prodigiosin from *S. marcescens* HMS.

Previous studies have demonstrated that prodigiosin exhibits considerable stability under various conditions. When dissolved in acetone, the pigment remained stable even at 100 °C for 1 h (Vaidyanathan et al. 2012). At a moderate temperature of 25 °C, over 80% of prodigiosin was retained after 25 days. Moreover, prodigiosin concentration in acetone remained above 98% after 30 days at 4 °C, with the stability of acetone extracts being approximately 2% higher than that of ethanol extracts under the same conditions. Considering that prodigiosin can be stored at low temperatures, storage at 4 °C allows the pigment to be preserved safely in acetone for extended periods (Park et al. 2012).

*S. marcescens* has been reported to produce significant levels of prodigiosin, with variations in the timing of maximum pigment production. According to Srimathi et al. (2017) and Shete et al. (2023), substantial prodigiosin accumulation was observed after 96 h of incubation. In contrast, Xia et al. (2018) noted that the cells entered the stationary phase at 24 h, with maximum pigment production occurring by 36 h. These findings suggest that prodigiosin synthesis by *S. marcescens* is influenced by strain-specific or culture condition-dependent factors, leading to differences in the timing of maximal pigment yield.

Numerous studies have also highlighted the impact of culture and environmental factors on bacterial pigment production (Bhagwat and Padalia 2020; Han et al. 2021; Aman Mohammadi et al. 2022). This study provides new insights into how physical culture conditions influence pigment production and distribution. In the present study, optimal prodigiosin production (6.98 mg/L) was achieved in LB medium at pH 7 when cultures were incubated at 30 °C under shaking conditions (150 rpm). These findings are consistent with previous reports highlighting the influence of culture medium and incubation conditions on prodigiosin biosynthesis. For instance, a previous study reported that nutrient broth (NB) incubated at 30 °C resulted in a maximum prodigiosin yield of 15 mg/L (Paul et al. 2024). Similarly, another investigation demonstrated that *S. marcescens* exhibited its highest prodigiosin production in nutrient agar (NA) medium during the 6th–7th day of incubation, reaching up to 72 mg/L (Koyun et al. 2022).

Earlier research has largely focused on intracellular prodigiosin pigment accumulation, with little attention given to the possibility of extracellular prodigiosin pigment release. In contrast, our results show that static conditions favor the accumulation of prodigiosin pigment outside the cells, while shaking enhances intracellular prodigiosin pigment levels. The enhanced extracellular release under static conditions therefore represents a potentially novel characteristic of the *S. marcescens* HMS strain. This observation suggests that culture agitation may affect not only pigment synthesis but also its localization or transport mechanisms. Few studies have investigated the effect of static culture conditions on *S. marcescens*, and these have generally reported low or no intracellular prodigiosin production (Shaikh 2016; Shaba et al. 2017).

The commercial applicability of prodigiosin has been restricted due to its high production costs, which are partially attributable to the costly growth media. Therefore, finding an inexpensive substrate is an efficient means of lowering prodigiosin production expenses. Significant amounts of protein, dietary fiber, fatty acids, and minerals can still be found in some agro-industrial wastes and by-products. These wastes and by-products have been suggested as low-cost substitutes for carbon and nitrogen sources, either whole or in part, for the production of microbial metabolites. This will help to reduce pollution in the environment and reprocess these by-products to raise their market value (Campos et al. 2020; Han et al. 2021). For instance, sweet potato peels have been reported to contain 76.4–77.0% carbohydrates, 22.3–22.4% fibers, 6.40–6.49% proteins, and 2.33–2.65% fats, indicating their potential as a nutrient-rich substrate for microbial metabolite production (Maloney et al. 2014). Incorporating such by-products into fermentation strategies can enhance both the economic and environmental sustainability of prodigiosin production processes.

In contrast to previous studies that used potato or sweet-potato peels only in combination with additional nitrogen-rich supplements such as casein (Suryawanshi et al. 2014), the present work demonstrates that *S. marcescens* HMS can efficiently produce prodigiosin using these peels as the sole carbon and nitrogen sources. Eliminating the need for external nutrient supplementation enhances the cost-effectiveness and simplicity of the fermentation process.

Environmental stresses, such as limited oxygen under static incubation or nutrient restriction from using potato and sweet potato peels, can disrupt *S. marcescens* membrane integrity. This triggers the accumulation of misfolded proteins or peptidoglycan fragments in the periplasm, promoting vesicle formation. Consequently, prodigiosin is found not only in intracellular granules but also in extracellular and cell-associated vesicles. Extracellular vesicle formation thus represents a bacterial strategy to cope with stress by removing misfolded proteins and facilitating pigment release (Hamada and Mohamed 2024; Pandey et al. 2024).

Following pigment production, optimization, and extraction, the compound was further purified using column chromatography and subsequently analyzed through various characterization methods. TLC analysis of the purified compound yielded chromatographic characteristics consistent with prodigiosin, supporting successful purification and aligning with previously published reports (Bhagwat and Padalia 2020).

The strong correspondence between the HPLC retention behavior of the purified pigment and that of standard prodigiosin supports its identification as prodigiosin. The appearance of a single dominant peak further indicates a high degree of chemical purity and sample homogeneity.

The FTIR profile of the isolated pigment is consistent with previously reported prodigiosin spectra, with characteristic bands, such as the strong absorption around 1639 cm⁻^1^ attributed to C = N stretching of the pyrrole ring, supporting its identity as a prodigiosin derivative (Halder et al. 2020). Additional features suggest the presence of amine and hydroxyl groups, providing further evidence of the pigment’s structural similarity to known prodigiosin compounds (Halder et al. 2020; Keekan et al. 2020; Zhao et al. 2021).

GC–MS analysis confirmed that the red pigment extracted from *S. marcescens* HMS has a molecular mass corresponding to the molecular formula C_20_H_25_N_3_O, with a molecular ion peak at 323 m/z, which is consistent with prodigiosin reported in previous studies (Li et al. 2021; Hamada and Mohamed 2024). The fragmentation pattern obtained from the mass spectrum further supports this identification, as the major fragment ions align with the characteristic cleavage pathways of the prodigiosin backbone.

Previous studies have demonstrated that prodigiosin exhibits a broad spectrum of biological activities, including cytotoxic effects against various cancer cell lines, as well as potent antibacterial and antifungal properties. Additionally, emerging evidence suggests its potential antidiabetic activity. These multifaceted bioactivities position prodigiosin as a promising candidate for further development in pharmaceutical applications, and potentially in food industry (Elghali et al. 2024; El-Sofany et al. 2025; Rafiq et al. 2025).

The antibacterial activity of prodigiosin observed against *B. subtilis* is consistent with previous reports demonstrating its efficacy against certain Gram-positive bacteria (Gulani et al. 2012; Ibrahim et al. 2014; Yip et al. 2021). Earlier investigations using the disc diffusion assay assessed the antibacterial potential of prodigiosin against a panel of six bacterial strains, comprising three Gram-positive (*MRSA, Staphylococcus aureus,* and *Enterococcus faecalis*) and three Gram-negative (*Escherichia coli, Salmonella Typhimurium,* and *Pseudomonas aeruginosa*) species. At a concentration of 500 μg/μL, prodigiosin effectively inhibited three Gram-positive strains (*MRSA, S. aureus,* and *E. faecalis*) and one Gram-negative strain (*E. coli*), producing inhibition zones of 21 ± 0.00 mm, 22 ± 0.33 mm, 20 ± 0.33 mm, and 27 ± 0.82 mm, respectively (Yip et al. 2021). The prodigiosin’s lipophilic nature, attributed to the presence of a monopyrrole C-ring chain, may contribute to its reduced efficacy against Gram-negative bacteria, which possess an outer membrane containing lipopolysaccharides that impede the penetration of lipophilic compounds (Pandey et al. 2004; Darshan and Manonmani 2016).

Conversely, several studies have reported that prodigiosin exhibits broad-spectrum antibacterial activity against both Gram-positive and Gram-negative bacteria (Arivizhivendhan et al. 2018; Hamada and Mohamed 2024; Rafiq et al. 2025). Consistent with these findings, Rafiq et al. (2025) reported that prodigiosin, at a concentration of 1,000 μg/mL, exhibited higher antibacterial activity against *E. coli* (28.2 ± 0.57 mm) compared with *B. subtilis* (23.58 ± 0.60 mm).

Prodigiosin exhibited cytotoxic activity against both HepG2 and MCF-7 cell lines, with a more pronounced effect observed on liver cancer (HepG2) cells. These findings align with previous research demonstrating that prodigiosin possesses potent antitumor properties across more than 60 cancer cell lines (Paul et al. 2022). Jardak et al. (2022) reported that prodigiosin produced by *Serratia* sp. C6LB exhibited IC_50_ values of 6.7 μg/mL and 16 μg/mL against the MDA-MB231 and MCF-7 breast cancer cell lines, respectively. Furthermore, a previous study showed that both prodigiosin and prodigiosin-conjugated silver nanoparticles exhibited significant cytotoxic effects against HepG2 cells, with IC_50_ values of 44.834 μg/mL and 29.8548 μg/mL, respectively (El-Batal et al. 2018). This suggests that conjugation with nanoparticles may enhance the cytotoxic potency of prodigiosin, representing a potential direction for future research.

α-Glucosidase inhibitors have the potential to serve as key therapeutic agents in the control and treatment of type 2 diabetes mellitus, which remains a major global public health issue (Nguyen et al. 2019). In the current investigation, prodigiosin demonstrated a moderate inhibitory effect on α-glucosidase, suggesting its potential as a natural antidiabetic compound. These results indicate that while prodigiosin does exhibit α-glucosidase inhibitory activity, its potency is considerably lower than that of acarbose. This contrasts with findings by Tran et al. (2021), who reported a 99% inhibition at 50 µg/mL and an IC_50_ of 0.0183 µg/mL for prodigiosin, suggesting a higher efficacy.

## Conclusion

*S. marcescens* HMS, identified by 16S rRNA sequencing and deposited in GenBank (PP456732.1), efficiently produces prodigiosin under optimized conditions. Acetone was the most effective solvent for extraction, and LB medium at pH 7 and 30 °C with 150 rpm shaking yielded maximum intracellular pigment production. Notably, under static conditions, prodigiosin was secreted extracellularly. The use of low-cost substrates, such as sweet potato peels, demonstrated promising extracellular prodigiosin production. Purification and characterization via TLC, HPLC, FTIR, and GC–MS confirmed the identity and high purity of prodigiosin (C_20_H_25_N_3_O, 323 m/z). The purified pigment exhibited antimicrobial activity against *B. subtilis*, cytotoxic effects against HepG2 and MCF-7 cells, and α-glucosidase inhibitory activity, highlighting its potential as a bioactive compound for pharmaceutical and biomedical applications.

Future perspectives include exploring low-cost agricultural substrates for sustainable, large-scale production, investigating prodigiosin conjugation with nanoparticles to enhance cytotoxic potency, evaluating structural modifications or formulations to improve stability and bioactivity, and studying potential synergistic effects with other antimicrobial or anticancer agents to broaden therapeutic applications.

## Supplementary Information

Below is the link to the electronic supplementary material.

Supplementary file1 (DOCX 353 KB)

## Data Availability

All data generated or analyzed during this study are included in this published article.

## Abbreviations

*S. marcescens*: *Serratia marcescens*
FDA: Food and Drug Administration
A_535_: Absorbance at 535 nm
rpm: Round per Minute
LB: Luria-Bertani medium
NB: Nutrient broth
YM: Yeast Malt medium
SM: Synthetic medium
OD: Optical density
v/v: Volume/Volume
UV-Vis: Ultraviolet-Visible
TLC: Thin Layer Chromatography
HPLC: High Performance Liquid Chromatography
FTIR: Fourier Transform Infrared Spectroscopy
GC-MS: Gas Chromatography and Mass Spectrometry
Rf: Retention factor
CFU: Colony forming unit
MTT: 3-(4,5-Dimethylthiazole-2-yl)-2,5 Diphenyl Tetrazolium Bromide
MCF-7: Human breast adenocarcinoma
HepG2: Human hepatocellular carcinoma
IC_50_: 50% Inhibitory concentration
DMSO: Dimethylsulfoxide
