# Supplementary material for: Bioproduction, characterization, and evaluation of the biological activities of prodigiosin from Serratia marcescens HMS
Source: World J Microbiol Biotechnol. 2026 Apr 25;42(5):234. doi: 10.1007/s11274-026-04936-8 (PMC13109127; doi:10.1007/s11274-026-04936-8)
Supplement: Supplementary file 1 — Supplementary file1 (DOCX 353 KB) [file 11274_2026_4936_MOESM1_ESM.docx]

**Supplementary materials**

**Bioproduction, characterization, and** **evaluation of the biological activities of prodigiosin from *Serratia marcescens* HMS**


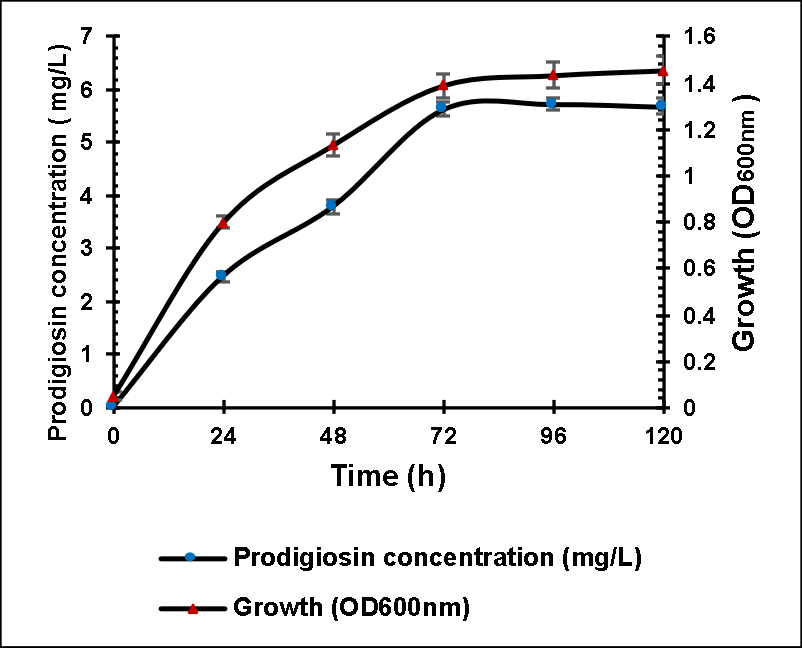


**Fig. S1** The growth and intracellular prodigiosin production by *S. marcescens* HMS at different incubation times on LB medium. Bars represent the mean values (± SD, n = 3) for each incubation time. One-way ANOVA demonstrated a significant difference in growth and prodigiosin production across the incubation periods (p < 0.001).

**Table S1** Summary of Optimization of Pigment Production by *S. marcescens* HMS, expressed as mean ± SD (n = 3).

| **Factors** | | **A_535_** | | **Intracellular prodigiosin concentration (mg/L)** | **Growth OD_600nm_** | **Degree of pigmentation** |
| --- | --- | --- | --- | --- | --- | --- |
|  |  | **Intracellular prodigiosin** | **Extracellular prodigiosin** |  |  |  |
| **Culture media** | **LB** | **1.253 ± 0.026** | **0.406 ± 0.027** | **5.73 ± 0.12** | **1.433 ± 0.051** | **0.87 ± 0.02** |
|  | **NB** | **0.640 ± 0.023** | **0.528 ± 0.024** | **2.93 ± 0.11** | **0.698 ± 0.046** | **0.92 ± 0.03** |
|  | **YM** | **0.045 ± 0.008** | **0.143 ± 0.015** | **0.21 ± 0.03** | **0.624 ± 0.055** | **0.07 ± 0.01** |
|  | **SM** | **0.053 ± 0.009** | **0.246 ± 0.017** | **0.24 ± 0.04** | **0.324 ± 0.048** | **0.16 ± 0.03** |
| **Temperature** | **25 °C** | **0.961 ± 0.032** | **0.356 ± 0.021** | **4.39 ± 0.14** | **1.196 ± 0.056** | **0.80 ± 0.03** |
|  | **30 °C** | **1.253 ± 0.028** | **0.406 ± 0.024** | **5.73 ± 0.13** | **1.433 ± 0.051** | **0.87 ± 0.02** |
|  | **35 °C** | **0.398 ± 0.019** | **0.240 ± 0.018** | **1.82 ± 0.08** | **0.774 ± 0.047** | **0.51 ± 0.03** |
| **pH** | **3** | **0.004 ± 0.001** | **0.082 ± 0.008** | **0.02 ± 0.01** | **0.045 ± 0.010** | **0.09 ± 0.02** |
|  | **4** | **0.002 ± 0.001** | **0.089 ± 0.007** | **0.01 ± 0.00** | **0.043 ± 0.015** | **0.05 ± 0.03** |
|  | **5** | **0.027 ± 0.004** | **0.087 ± 0.005** | **0.12 ± 0.02** | **0.098 ± 0.019** | **0.27 ± 0.05** |
|  | **6** | **0.973 ± 0.023** | **0.632 ± 0.019** | **4.45 ± 0.10** | **1.237 ± 0.054** | **0.79 ± 0.03** |
|  | **7** | **1.253 ± 0.028** | **0.406 ± 0.017** | **5.73 ± 0.13** | **1.433 ± 0.049** | **0.87 ± 0.02** |
|  | **8** | **1.032 ± 0.032** | **0.220 ± 0.013** | **4.72 ± 0.15** | **1.326 ± 0.061** | **0.78 ± 0.03** |
|  | **9** | **0.133 ± 0.004** | **0.262 ± 0.014** | **0.61 ± 0.02** | **0.348 ± 0.027** | **0.38 ± 0.01** |
|  | **10** | **0.001 ± 0.001** | **0.040 ± 0.005** | **0.01 ± 0.01** | **0.038 ± 0.008** | **0.03 ± 0.02** |
| **Agitation rate** | **0 rpm** | **0.589 ± 0.024** | **1.236 ± 0.034** | **2.69 ± 0.11** | **0.675 ± 0.048** | **0.87 ± 0.03** |
|  | **120 rpm** | **1.253 ± 0.031** | **0.406 ± 0.021** | **5.73 ± 0.14** | **1.433 ± 0.054** | **0.87 ± 0.02** |
|  | **150 rpm** | **1.526 ± 0.036** | **0.155 ± 0.018** | **6.98 ± 0.16** | **1.401 ± 0.057** | **1.09 ± 0.02** |

**Fig. S2** Effect of using peels of potato and sweet potato on *S. marcescens* HMS growth and the absorbance of extracellular prodigiosin at 535 nm. Bars represent mean ± SD (n = 3).

**
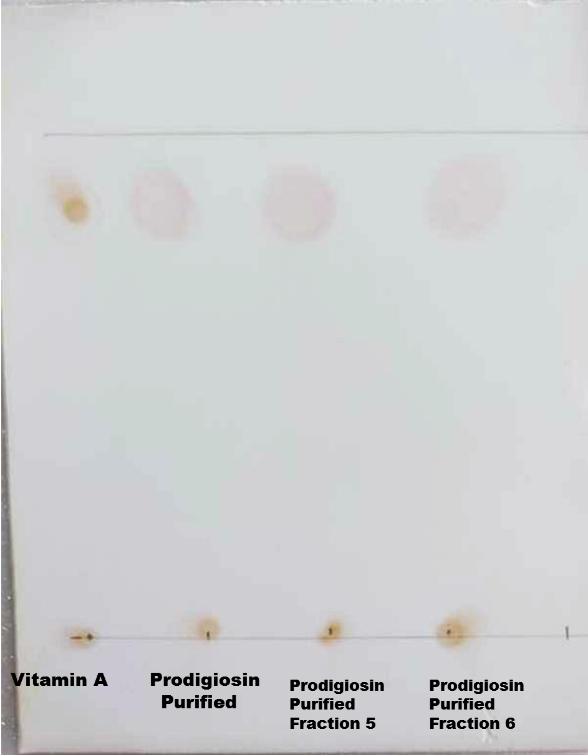
**

**Fig. S3** TLC analysis of the pigment’s fractions.


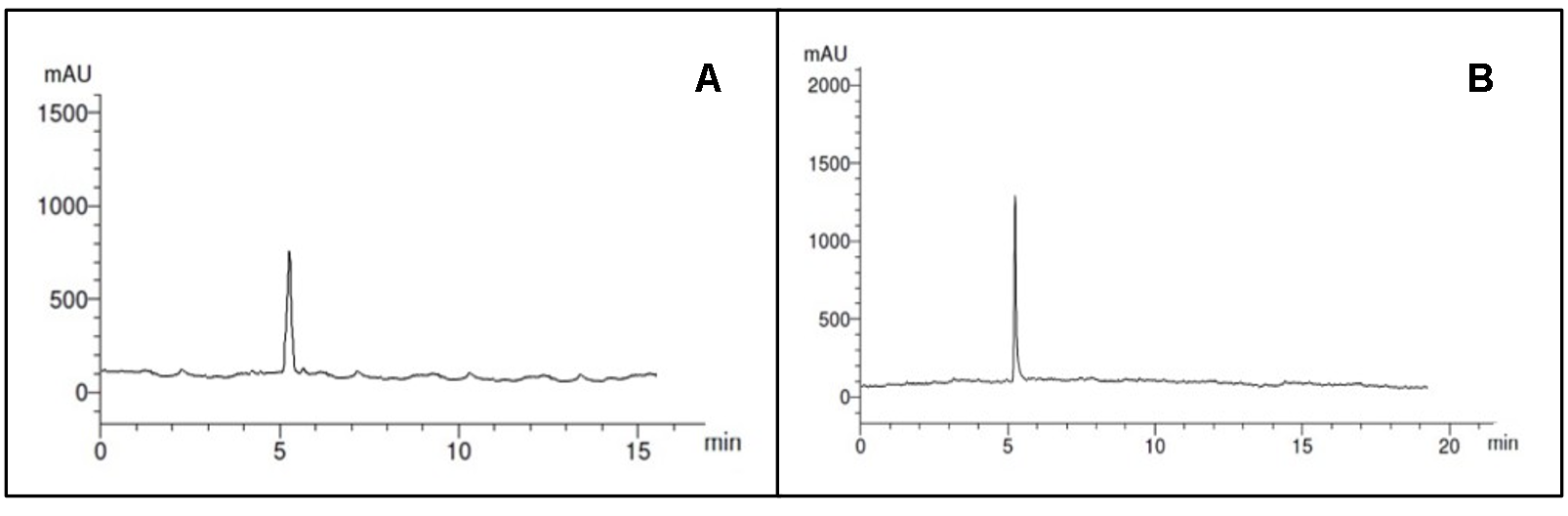


**Fig. S4** HPLC detection of prodigiosin. HPLC chromatogram of purified prodigiosin sample **(a)**, HPLC chromatogram of standard prodigiosin **(b)**.

**Table S2** GC–MS Profile of purified prodigiosin: Retention time, relative abundance, and major fragments

| **Peak no.** | **Retention time** | **Area %** | **Major fragments (m/z)** | **Peak no.** | **Retention time** | **Area %** | **Major fragments (m/z)** |
| --- | --- | --- | --- | --- | --- | --- | --- |
| **1** | 5.73 | 2.90 | 58, 100 | **19** | 69.48 | 3.61 | 73, 147, 221, 281 |
| **2** | 12.97 | 2.01 | 84, 126 | **20** | 70.04 | 0.91 | 73, 147, 207, 221, 281, 323 |
| **3** | 14.99 | 1.53 | 83, 140 | **21** | 70.26 | 0.66 | 73, 147, 207, 221, 281, 295, 323 |
| **4** | 17. 25 | 0.51 | 83, 96, 126, 140 | **22** | 72.46 | 3.96 | 59, 73, 126, 207, 281 |
| **5** | 32.69 | 1.40 | 191 | **23** | 73. 56 | 5.18 | 73, 147, 207, 22169, 281, 295, 323 |
| **6** | 36.51 | 0.40 | 82, 96 | **24** | 74.01 | 2.33 | 69, 73, 147, 207, 221, 281, 295, 323 |
| **7** | 44.33 | 0.34 | 159, 177, 205 | **25** | 74.06 | 1.67 | 69, 73, 147, 207, 221, 281, 295 |
| **8** | 46.19 | 1.14 | 175, 205 | **26** | 74.27 | 2.55 | 69, 73, 147, 207, 221, 281 |
| **9** | 47.32 | 0.61 | 147, 277 | **27** | 75.76 | 3.47 | 67, 95, 121, 147, 207, 281 |
| **10** | 47.59 | 3.21 | 74, 87, 143 | **28** | 76.24 | 6.22 | 81, 95, 207, 221, 281 |
| **11** | 49.01 | 1.10 | 177, 205, 220 | **29** | 76.68 | 1.51 | 95, 207, 221, 281 |
| **12** | 50.18 | 1.27 | 55, 83, 97, 250, 252, 281 | **30** | 77.05 | 5.74 | 73, 147, 207, 221, 281, 295, 323 |
| **13** | 53.85 | 1.11 | 74, 87, 143, 255, 298 | **31** | 78.57 | 1.48 | 81, 147, 207, 221, 281, 295, 323 |
| **14** | 61.83 | 0.99 | 57, 73, 147, 190, 283 | **32** | 80.20 | 5.53 | 73, 147, 207, 221, 281, 295, 323 |
| **15** | 64.23 | 0.83 | 73, 147, 221, 281 | **33** | 83.14 | 5.02 | 73, 147, 207, 221, 281, 295, 323 |
| **16** | 64.27 | 0.64 | 73, 147, 207, 221, 281 | **34** | 86.51 | 4.31 | 73, 147, 207, 221, 281, 295, 323 |
| **17** | 65.74 | 1.59 | 73, 117, 147, 207, 221, 281 | **35** | 87.87 | 0.98 | 73, 147, 207, 281, 308 |
| **18** | 66.56 | 1.33 | 149, 167, 279 | **36** | 90.83 | 3.84 | 73, 147, 207, 221, 281, 295, 323 |

**Table S3** Mean diameters of inhibition zones (mm) produced by purified prodigiosin and positive controls against a range of pathogenic microorganisms. Values are presented as mean ± SD, (NA: no activity).

| **Tested microorganisms** | **Purified prodigiosin** | **Positive control** |
| --- | --- | --- |
| **Gram positive bacteria** |  | **Gentamycin** |
| *Bacillus subtilis* RCMB 015 (1) NRRL B-543 | 12 ± 0.41 | 26 ± 0.68 |
| *Staphylococcus aureus* ATCC 25923 | NA | 24 ± 0.60 |
| **Gram negative bacteria** |  | **Gentamycin** |
| *Escherichia coli* ATCC 25922 | NA | 30 ± 1.21 |
| *Proteus vulgaris* RCMB 004 (1) ATCC 13315 | NA | 25 ± 0.42 |
| **Fungi** |  | **Ketoconazole** |
| *Aspergillus fumigatus* RCMB 002008 | NA | 17 ± 0.82 |
| *Candida albicans* RCMB 005003 (1) ATCC 10231 | NA | 20 ± 0.57 |
